# A Minimally Invasive Endoscopic Surgery for Infectious Spondylodiscitis of the Thoracic and Upper Lumbar Spine in Immunocompromised Patients

**DOI:** 10.1155/2015/780451

**Published:** 2015-07-26

**Authors:** Hsin-Chuan Chen, Teng-Le Huang, Yen-Jen Chen, Hsi-Kai Tsou, Wei-Ching Lin, Chih-Hung Hung, Chun-Hao Tsai, Horng-Chaung Hsu, Hsien-Te Chen

**Affiliations:** ^1^Department of Orthopedic Surgery, Show Chwan Memorial Hospital, No. 542, Section 1, Zhongshan Road, Changhua City, Changhua County 500, Taiwan; ^2^Department of Orthopedics, Tainan Municipal An-Nan Hospital-China Medical University, No. 66, Section 2, Changhe Road, Annan District, Tainan City 709, Taiwan; ^3^Department of Bioscience Technology, College of Science, Chung Yuan Christian University, No. 200 Chung Pei Road, Chung Li District, Taoyuan City 320, Taiwan; ^4^Department of Sports Medicine, College of Health Care, China Medical University, No. 91, Hsueh-Shih Road, North District, Taichung 404, Taiwan; ^5^Department of Orthopedic Surgery, China Medical University Hospital, No. 2 Yuhder Road, North District, Taichung 404, Taiwan; ^6^School of Medicine, China Medical University, No. 91 Hsueh-Shih Road, North District, Taichung 404, Taiwan; ^7^Functional Neurosurgery Division, Neurological Institute, Taichung Veterans General Hospital, 1650 Taiwan Boulevard Section 4, Taichung 407, Taiwan; ^8^Department of Early Childhood Care and Education, Jen-Teh Junior College of Medicine, Nursing and Management, No. 79-9 Sha-Luen Hu, Xi-Zhou Li, Hou-Loung Town, Miaoli County 356, Taiwan; ^9^Department of Radiology, China Medical University Hospital, No. 2 Yuhder Road, North District, Taichung 404, Taiwan; ^10^China Medical University, No. 91 Hsueh-Shih Road, North District, Taichung 404, Taiwan; ^11^School of Chinese Medicine, College of Chinese Medicine, China Medical University, No. 91 Hsueh-Shih Road, North District, Taichung 404, Taiwan

## Abstract

This study evaluates the safety and effectiveness of computed tomography- (CT-) assisted endoscopic surgery in the treatment of infectious spondylodiscitis of the thoracic and upper lumbar spine in immunocompromised patients. From October 2006 to March 2014, a total of 41 patients with infectious spondylodiscitis underwent percutaneous endoscopic surgery under local anesthesia, and 13 lesions from 13 patients on the thoracic or upper lumbar spine were selected for evaluation. A CT-guided catheter was placed before percutaneous endoscopic surgery as a guide to avoid injury to visceral organs, major vessels, and the spinal cord. All 13 patients had quick pain relief after endoscopic surgery without complications. The bacterial culture rate was 77%. Inflammatory parameters returned to normal after adequate antibiotic treatment. Postoperative radiographs showed no significant kyphotic deformity when compared with preoperative films. As of the last follow-up visit, no recurrent infections were noted. Traditional transthoracic or diaphragmatic surgery with or without posterior instrumentation is associated with high rates of morbidity and mortality, especially in elderly patients, patients with multiple comorbidities, or immunocompromised patients. Percutaneous endoscopic surgery assisted by a CT-guided catheter provides a safe and effective alternative treatment for infectious spondylodiscitis of the thoracic and upper lumbar spine.

## 1. Introduction

In recent years, the incidence of infectious spondylodiscitis has increased due to vast improvements in medical care and prolonged life expectancies. The condition is associated with advanced age, intravenous drug use, immunocompromised status, and significant medical comorbidities [1]. Identifying the causative pathogen is the key to treatment. Computed tomography- (CT-) guided biopsy and drainage are the standard procedure for identifying causative pathogens. However, the pathogen-identification rate varies among studies [2–4]. Surgical intervention is indicated if neurological deficit, progressive deformity, failure to respond to conservative treatment, or the need to obtain specimens to identify causative pathogens is present. However, traditional anterior debridement and reconstruction with or without posterior instrumentation are associated with high rates of morbidity and mortality, especially in elderly immunocompromised patients and patients with multiple comorbidities. Percutaneous endoscopic discectomy, debridement, and drainage provide a minimally invasive surgical choice for the treatment of infectious spondylodiscitis [4–6]. This method provides adequate debridement and fast pain relief and has a relatively high pathogen-identification rate [4, 7, 8]. However, in the upper lumbar and thoracic spine, percutaneous endoscopy is associated with visceral organ damage, major vessel injury, and the spinal cord injury, which limits the use of this procedure in these areas [9].

A CT-guided catheter was placed before percutaneous endoscopic surgery as a guide to avoid injury to visceral organs, major vessels, and the spinal cord. We analyzed the clinical outcomes, inflammatory parameters, and radiographic findings for 13 lesions that occurred on thoracic or upper lumbar spine.

## 2. Materials and Methods

### 2.1. Patient Population

**Figure 1:**
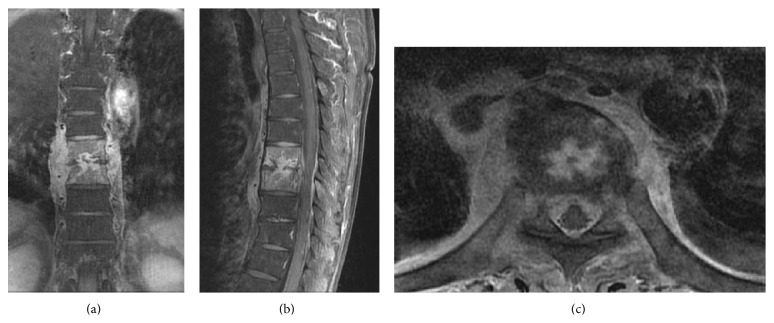
MRI evidence of T8-T9 infectious spondylodiscitis in patient number 13 in (a) coronal, (b) sagittal, and (c) axial view. T1-weighted signal with gadolinium contrast of spine MRI showed increased signal at the T8 and T9 vertebral bodies and also ring enhancement of the destroyed T8-T9 intervertebral disc.

From October 2006 to March 2014, a total of 41 patients with infective spondylodiscitis underwent percutaneous endoscopic surgery with local anesthesia and 13 patients' lesions on the thoracic or upper lumbar spine were selected for evaluation (Table 1). A CT-guided angiographic catheter was placed before percutaneous endoscopic surgery as a guide to avoid injury to visceral organs, major vessels, and the spinal cord. Of the 13 patients evaluated, 5 were men and 8 were women. Their mean age was 65.6 years (range, 49–84 years). The affected level ranged from T11-T12 to L1-L2 in 11 cases, T8-T9 in 1 case, and T9-T10 in the other case. All patients underwent plain film radiography and enhanced magnetic resonance imaging (MRI) of the involved spine, which revealed evidence of infectious spondylodiscitis (Figure 1). Most patients had high inflammatory markers (C-reactive protein (CRP) and erythrocyte sedimentation rate (ESR)) and complained of severe back pain. They also had a variety of comorbidities, including renal failure, heart failure, rheumatic arthritis, liver cirrhosis, polycystic liver posttransplantation, and diabetes (Table 1).

### 2.2. Surgical Procedures

**Figure 2:**
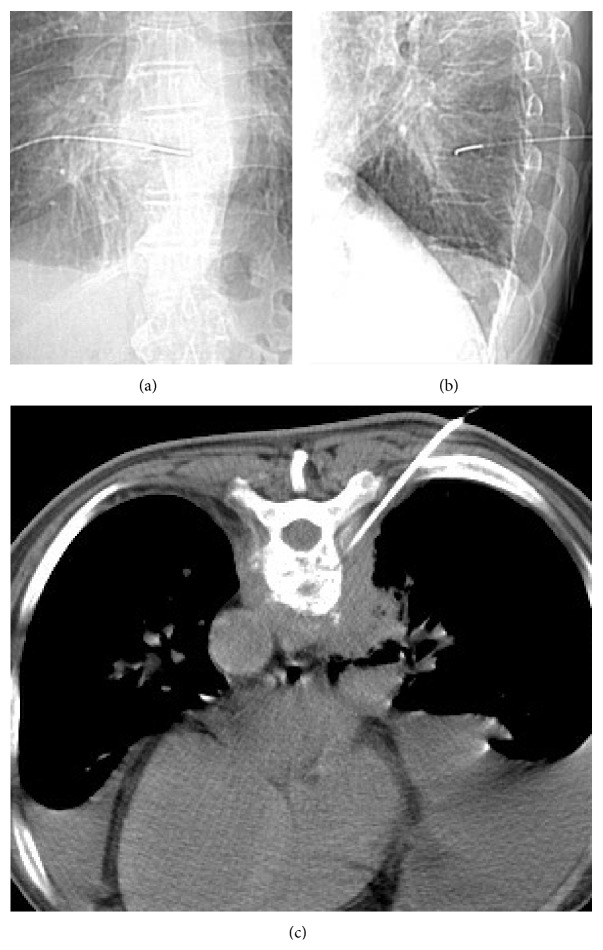
CT-guided catheterization in T8-T9 intervertebral disc: (a) anteroposterior, (b) lateral projection of the catheter during the procedure, and (c) axial illustration of the catheter in the intervertebral disc with CT-guided procedure.

The CT-guided biopsy and catheter placement were performed by an experienced radiologist on the day of or the day before the scheduled percutaneous endoscopic surgery (Figure 2). The patient was positioned prone, and local anesthesia with 2% lidocaine was injected into the area of needle insertion. A 6-in-long number 11G-wide multiple side hole bone puncture needle was inserted into the lesion site with CT guidance. A J guidewire was then inserted via the bone biopsy needle. Finally, a number 5 Fr C1 angiographic catheter (Cook, Bloomington, USA) was inserted along the J guidewire and left in the infective area after guidewire removal. The specimen obtained during the procedure was sent for bacterial, tuberculosis (TB), and fungal cultures and pathologic analysis.

**Figure 3:**
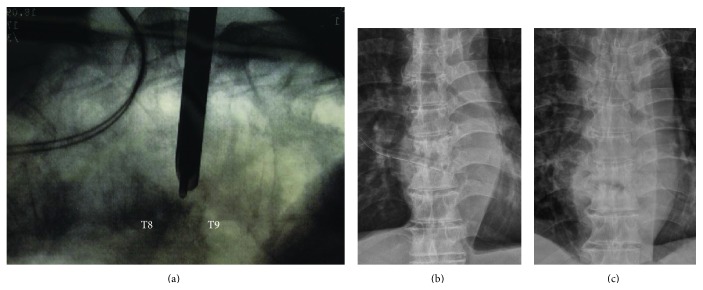
(a) Intraoperative fluoroscopic image demonstrated the working sheath and endoscope within the intervertebral disc space, (b) immediate postoperative AP view demonstrated a 1/4 inch drainage tube in the intervertebral space, and (c) postoperative 3-month AP view revealed partially united T8-T9 vertebral body.

Percutaneous endoscopic surgery was then performed after placement of the CT-guided angiographic catheter. The patient was positioned prone on a spine-operating table with the abdomen hanging free. The patient was under intravenous pain control but was kept awake during the endoscopic surgery so that he or she could respond well when the dura or nerve roots were irritated. Local anesthesia was also performed with 2% lidocaine around the area of endoscopic insertion. A percutaneous endoscopic guidewire was inserted directly through the CT-guided number 5 Fr C1 angiographic catheter and advanced slowly with the assistance of fluoroscopy to ensure that the wire was targeting the infective area without penetrating the angiographic catheter wall and injuring any related structures. After the guidewire was set in the infected area, the number 5 Fr C1 angiographic catheter was then removed. A dilator was inserted along the endoscopic guidewire, and the position was again checked under fluoroscopy. The infected disc and vertebra were harvested for bacterial, fungal, and TB cultures by using endoscopic microrongeurs and microscissors before starting irrigation. After adequate tissue sampling for culture, radical debridement, sequestrectomy, and irrigation could be performed with the aid of direct endoscope vision and fluoroscopy. The Surgitron, a high-voltage bipolar probe (Ellman Innovations, New York, USA), was used for thermocoagulation of infected tissue and bleeders. All the operating instruments and endoscopic systems were supplied by Richard and Wolf (Knittlingen, Germany). The high-resolution endoscope has a diameter of 8 mm with a 4.1 mm intraendoscopic working channel. The angle of vision is 25°. The working sheath has an 8.0 mm outer diameter and a beveled opening, both of which enable the creation of visual and working fields in an area without a clear, anatomically preformed cavity. More than 4 L normal saline with 1 g cefazolin in each liter was used for pressured irrigation and drainage of infected materials and pus. A 1/4 in drainage tube was left in the infected area at the end of surgery for further drainage of infective materials, pus, and exudates (Figure 3).

### 2.3. Postoperative Care

The 1/4 in drainage tube was left in place for at least 7 days until the daily drainage amount was less than 5 mL. Effective antibiotics were administered intravenously for patients with known causative pathogens before surgery. For patients with unknown pathogens, empirical antibiotics were administered immediately after surgery. These were switched to specific antibiotics after identification of the causative pathogen was made from intraoperative tissue or pus culture. Intravenous antibiotics were used for 4 to 6 weeks according to follow-up inflammatory markers. The patients were then switched to oral antibiotics and discontinued when the inflammatory markers were within a normal range. The patient remained in bed for 2 weeks after surgery. A rigid thoracolumbar spinal orthosis was then used for ambulation. The orthosis was used until radiographs showed evidence of bone union or prominent syndesmophyte formation along the anterior lateral aspect of the infected level.

### 2.4. Clinical Evaluation

Preoperative clinical symptoms and signs were recorded. Pain was evaluated with a visual analogue scale (VAS; 0–10) before and after surgery at 1 month, 3 months, and 6 months. The ESR and CRP levels were checked before surgery and every week after surgery. Spine plain radiography was performed immediately after surgery and at 1 month, 3 months, 6 months, and 1 year after surgery. Any evidence of spinal kyphotic deformity due to infectious spondylodiscitis was recorded.

## 3. Results

All patients had prominent back pain (VAS = 9.23) before surgery, and all reported quick pain relief (VAS = 2.31) after endoscopic surgery and antibiotic treatment (Table 4). The causative pathogens were identified in 10 patients (77% culture rate) postoperatively (Table 2), and effective antibiotics were administered according to the sensitivity test of isolated pathogens. The blood culture result of patient 7 was methicillin-sensitive* Staphylococcus aureus* infection, but the results of the endoscopic specimen were negative. Oxacillin was used for infection control according to the sensitivity test report of blood culture. The other 2 patients had no known culture results, and empirical antibiotics were maintained until the inflammatory parameters returned to normal ranges. In patient 9, anti-TB medication was maintained for 9 months according to TB treatment protocol. The ESR and CRP levels of all patients decreased significantly after endoscopic surgery and 3 months of intravenous and oral antibiotic treatment. No patient had relapse of spinal infection at the treated level during the follow-up period (Table 3).

**Figure 4:**
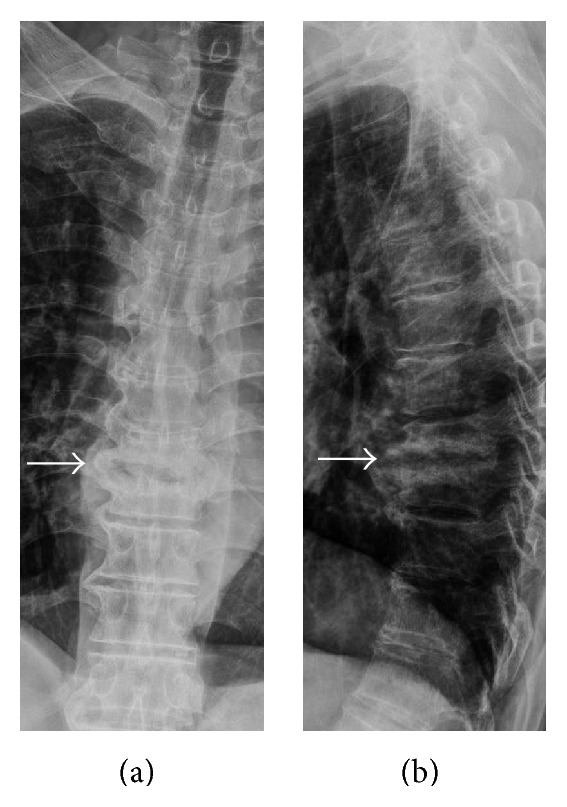
The plain films 6 months after operation. (a) AP view and (b) lateral view revealed prominent syndesmophyte formation at anterior and lateral aspects of infective area without significant kyphotic deformity.

No surgery-related complications were noted during the follow-up period (average, 42.46 months; range, 8–70 months). No recurrence of infection developed in these patients, and no open spinal surgery was needed. Deformity of the spine was evaluated from plain film radiographs obtained before and after surgery and at the last follow-up. The kyphotic angle was measured as the angle between the upper end plate of the first vertebral body above the involved level and the lower end plate of the first vertebral body below the involved level. Only 1 patient had a kyphotic change greater than 10°. The other 12 patients had no significant changes in spinal deformity (Table 5; Figure 4).

## 4. Discussion

Infectious spondylodiscitis is an increasingly prevalent disease, especially in elderly or immunocompromised patients or those with medical comorbidities. Conservative treatment consisting of antibiotic administration and bed rest is the standard choice in cases of mild infection. CT-guided biopsy is a less invasive procedure used to obtain specimens for pathogen identification. Drainage function also contributes to infection control. However, the rate of pathogen identification varies widely, from 36% to 91% [2–4, 10–12], and the rate of successful infection control is also unsatisfactory. When conservative treatment fails, the treatment approach shifts to open surgery. However, traditional anterior debridement and reconstruction with or without posterior instrumentation are a major operation with a high rate of postoperative complications, especially in immunocompromised patients with many comorbid diseases or in the elderly.

Percutaneous endoscopic discectomy was first applied for lumbar disc herniation in the 1980s and now is a well-established surgical procedure. This procedure has also been used to treat infectious spondylodiscitis in recent years and has proved to be as effective as open surgery for infection control [4, 6, 8, 13]. However, percutaneous endoscopic surgery for infectious spondylodiscitis has been limited to cases with lower lumbar spine infection for reasons of operative safety [7]. Percutaneous endoscopic surgery carries risks of injury to the surrounding visceral organs, the spinal cord, and major vessels of the thoracic and upper lumbar spine when used to treat lesions above the L2 level. In this study, a combination of a CT-guided angiographic catheter and percutaneous endoscopic surgery was found to be effective and safe for the treatment of infectious spondylodiscitis of the upper lumbar and thoracic spine. CT provides real-time images that can be used to insert the guidewire and catheter, which helps prevent injury to the spinal cord, major vessels, or visceral organs. Once the endoscope is inserted safely along the CT-guided catheter, percutaneous endoscopic surgery can be performed safely to carry out radical debridement or obtain a sufficient specimen culture, and a large-diameter drainage tube can be left in the infected level. Infection was well controlled with antibiotics in all patients, regardless of the culture results. No complications occurred during the operation or follow-up period. Moreover, no open surgery was needed in these patients, whereas anterior debridement and fusion surgery was previously performed in 19% to 25% of patients [4, 6].

In view of the risks of general anesthesia and traditional major surgery for infectious spondylodiscitis of the thoracic and upper lumbar spine, minimally invasive endoscopic surgery represents a good alternative treatment for immunocompromised patients. Percutaneous endoscopic surgery assisted by a CT-guided catheter is a safe and effective operation using local anesthesia and a small incision wound (<1 cm).

## 5. Conclusion

Percutaneous endoscopic discectomy, debridement, irrigation, and drainage with the assistance of a CT-guided catheter are a safe and effective alternative treatment for infectious spondylodiscitis of the thoracic and upper lumbar spine. It prevents many complications normally associated with percutaneous endoscopic surgery in the thoracic and upper lumbar area. Endoscopic surgery not only has a high rate of the causative-pathogen identification but also provides good infection control and pain relief, even if causative pathogen cannot be identified. The safe and minimally invasive nature of this procedure broadens the application of operative treatment for infectious spondylodiscitis, even in the thoracic and upper lumbar level, and prevents progressive deformity when prolonged antibiotic treatment is used. We have more confidence when treating patients in poor health, immunocompromised or elderly patients, and can now provide a more promising and safe treatment strategy. Motor weakness of the lower limbs may be the only contraindication for this procedure.

## Figures and Tables

**Table 1:** Patient demographic data.

Patient number	Age	Gender	Level	Neurological deficit	Associated medical illness
1	71	F	L1-2	Frankle D	Uremia, CHF, RHD
2	60	M	T11-12	Frankle D	CAD, DM, CHF, asthma
3	67	F	T12	Nil	Uremia, DM
4	65	F	L1-2	Nil	HTN, RA
5	55	F	T11-12	Nil	DM, HTN
6	73	F	T12-L1	Mild sensory deficit	DM
7	71	F	T11-T12	Nil	DM, liver cirrhosis, burst fracture T11 vertebra
8	71	M	L1	Frankle D	None
9	84	F	T9-10	Frankle D	HTN, CHF, PSVT
10	63	M	T12-L1-2	Mild sensory deficit	Liver cirrhosis, asthma
11	52	M	L1-2	Nil	BPH
12	49	F	L1-2	Nil	Polycystic liver, kidney s/p liver transplantation
13	72	M	T8-9	Mild sensory deficit	DM
Average	**65.6**				

Standard deviation	**9.73**				

CHF: congestive heart failure, RHD: rheumatic heart disease, CAD: coronary artery disease, DM: diabetes mellitus, HTN: hypertension, RA: rheumatoid arthritis, PSVT: paroxysmal supraventricular tachycardia, and BPH: benign prostate hypertrophy.

**Table 2:** Surgical procedures.

Patient	Level	Procedures	Bacteria culture
1^∗^	L1-L2	CT-guided catheter + PEDD	*Delftia acidovorans *
2^∗^	T11-T12	CT-guided catheter + PEDD	No growth
3	T12	CT-guided catheter + PEDD	No growth
4	L1-L2	CT-guided catheter + PEDD	*Escherichia coli* (ESBL)
5	T11-T12	CT-guided catheter + PEDD	*Staphylococcus aureus *
6	T12-L1	CT-guided catheter + PEDD	*Staphylococcus aureus* (MRSA)
7	T11-T12	CT-guided catheter + PEDD	No growth
8^∗^	L1	CT-guided catheter + PEDD	*Streptococcus anginosus *
9	T9-10	CT-guided catheter + PEDD	*Mycobacteria tuberculosis* complex
10	T12-L1-2	CT-guided catheter + PEDD	*Klebsiella pneumoniae *
11	L1-L2	CT-guided catheter + PEDD	*Staphylococcus aureus *
12	L1-L2	CT-guided catheter + PEDD	*Candida albicans, Ecoli *
13	T8-9	CT-guided catheter + PEDD	*Klebsiella pneumoniae *

PEDD: percutaneous endoscopic discectomy drainage.

^∗^Dying of other medical problems during next few years.

**Table 3:** ESR and CRP levels before and after surgery.

Patient number	ESR (mm/1 hr)				CRP (mg/dL)		
	Preop	Postop 1 m	Postop 3 m	Last F/U	Preop	Postop 1 m	Postop 3 m
1	51	67	20	37	6.62	0.38	2.84
2	76	18	21	13	22.16	0.15	0.18
3	115	86	78	72	32.11	2.70	0.60
4	79	41	9	8	4.96	0.45	0.03
5	108	71	25	21	6.33	0.31	0.08
6	123	115	64	36	23.72	1.24	0.55
7	112	93	86	84	1.44	0.18	0.29
8	75	42	14	7	18.19	2.80	0.95
9	53	28	18	28	6.61	1.94	0.75
10	125	85	37	30	0.19	0.07	0.06
11	92	16	2	2	7.45	0.23	0.12
12	108	74	50	9	4.84	0.70	0.11
13	104	12	11	11	16.53	0.25	0.35
Average	93.02	57.53	33.46	27.53	11.63	0.88	0.53

Standard deviation	25.00	33.44	27.51	25.26	9.85	0.98	0.75

**Table 4:** Back pain level before and after surgery.

Patient number	Visual analog scale (0–10)			Complication
	Preop	Postop 1 m	Postop 3 m	
1	10	5	2	Nil
2	9	4	2	Nil
3	10	5	3	Nil
4	10	4	1	Nil
5	10	5	4	Nil
6	10	5	3	Nil
7	10	4	3	Nil
8	9	5	1	Nil
9	9	4	3	Nil
10	10	5	4	Nil
11	8	1	1	Nil
12	8	3	2	Nil
13	7	3	1	Nil
Average	9.23	4.08	2.31	

Standard deviation	1.01	1.19	1.11	

**Table 5:** Changes in kyphosis angle (°).

Patient number	Level	Preoperative	Postoperative	Last F/U
1	L1-2	−6°	−2°	−1°
2	T11-12	10°	8°	10°
3	T12	16°	19°	19°
4	L1-2	2°	−2°	−1°
5	T11-12	6°	4°	1°
6	T12-L1	13°	13°	24°
7	T11-12	11°	14°	12°
8	L1	12°	5°	4°
9	T9-10	0°	3°	**3**°
10	T12-L1-2	−13°	−12°	−12°
11	L1-2	7°	8°	12°
12	L1-2	17°	16°	16°
13	T8-9	12°	14°	15°
Average		8.69	6.77	7.85

Standard deviation		6.68	8.72	9.91

Kyphosis angle was obtained by measuring the sagittal angles with the Cobb method.
